# Supplementary material for: Splice-Junction-Based Mapping of Alternative Isoforms in the Human Proteome
Source: Cell Rep. Author manuscript; Available in PMC 2020 Jan 15. (PMC6961840; doi:10.1016/j.celrep.2019.11.026)
Supplement: 2 [file NIHMS1546469-supplement-2.zip › DF1/PXD006675/VentricularSeptum/VentricularSeptum_18_TTN_VEAEPAEEVTIMEEK.pdf]

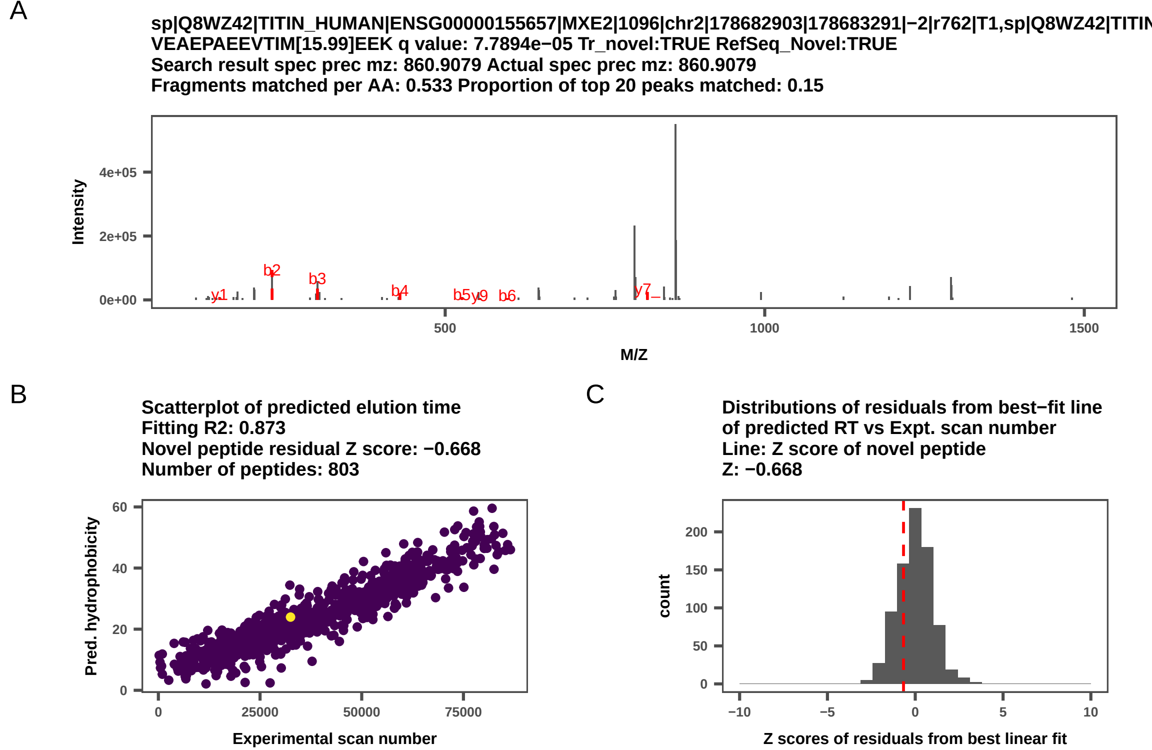
A
sp|Q8WZ42|TITIN_HUMAN|ENSG00000155657|MXE2|1096|chr2|178682903|178683291|−2|r762|T1,sp|Q8WZ42|TITIN
VEAEPAEEVTIM[15.99]EEK q value: 7.7894e−05 Tr_novel:TRUE RefSeq_Novel:TRUE
Search result spec prec mz: 860.9079 Actual spec prec mz: 860.9079
Fragments matched per AA: 0.533 Proportion of top 20 peaks matched: 0.15
Intensity
4e+05
2e+05
0e+00
y1
b2
b3
b4
b5
y9
b6
y7
500
1000
1500
M/Z
B
Scatterplot of predicted elution time
Fitting R2: 0.873
Novel peptide residual Z score: −0.668
Number of peptides: 803
Pred. hydrophobicity
60
40
20
0
0
25000
50000
75000
Experimental scan number
C
Distributions of residuals from best−fit line
of predicted RT vs Expt. scan number
Line: Z score of novel peptide
Z: −0.668
count
200
150
100
50
0
−10
−5
0
5
10
Z scores of residuals from best linear fit
